# Characterization of key information sections in informed consent forms posted on ClinicalTrials.gov

**DOI:** 10.1017/cts.2023.605

**Published:** 2023-08-14

**Authors:** Luke Gelinas, Walker Morrell, Tony Tse, Ava Glazier, Deborah A. Zarin, Barbara E. Bierer

**Affiliations:** 1 Advarra Inc., Columbia, MD, USA; 2 Division of Global Health Equity, Department of Medicine, Multi-Regional Clinical Trials Center of Brigham and Women’s Hospital and Harvard, Brigham and Women’s Hospital, Boston, MA, USA; 3 National Center for Biotechnology Innovation, National Library of Medicine, National Institutes of Health, Bethesda, MD, USA; 4 Brown University, Providence, RI, USA; 5 Department of Medicine, Harvard Medical School, Boston, MA, USA

**Keywords:** Informed consent, key information, common rule, regulations, ethics

## Abstract

**Introduction:**

Recent revisions to the US Federal Common Rule governing human studies funded or conducted by the federal government require the provision of a “concise and focused” key information (KI) section in informed consent forms (ICFs). We performed a systematic study to characterize KI sections of ICFs for federally funded trials available on ClinicalTrials.gov.

**Methods:**

We downloaded ICFs posted on ClinicalTrials.gov for treatment trials initiated on or after the revised Common Rule effective date. Trial records (*n* = 102) were assessed by intervention type, study phase, recruitment status, and enrollment size. The ICFs and their KI sections, if present, were characterized by page length, word count, readability, topic, and formatting elements.

**Results:**

Of the 102 trial records, 76 had identifiable KI sections that were, on average, 10% of the total length of full ICF documents. KI readability grade level was not notably different from other sections of ICFs. Most KI sections were distinguished by section headers and included lists but contained few other formatting elements. Most KI sections included a subset of topics consistent with the basic elements of informed consent specified in the Common Rule.

**Conclusion:**

Many of the KI sections in the study sample aligned with practices suggested in the preamble to the revised Common Rule. Further, our results suggest that some KI sections were tailored in study-specific ways. Nevertheless, guidelines on how to write concise and comprehensible KI sections would improve the utility and readability of KI sections.

## Introduction

Informed consent forms (ICFs) provide written information to individuals as part of a larger consent process aimed at enabling informed decision-making about research participation [1]. However, ICFs are often lengthy and written in complex language, potentially interfering with comprehension [2–7]. Among recent revisions to the US Federal Policy for the Protection of Human Subjects (the “Common Rule”), which applies to research conducted, supported, or otherwise subject to regulation by the federal government, is a provision aiming to facilitate understanding by requiring the inclusion of a brief “key information” (KI) section at the beginning of ICFs [8–10]. Specifically, for trials initiated on or after the compliance date (January 21, 2019), “informed consent must begin with a concise and focused presentation of the KI that is most likely to assist a prospective subject or legally authorized representative in understanding the reasons why one might or might not want to participate in the research” [10]. In addition, the recent notice of proposed rulemaking issued by the US Food and Drug Administration (FDA) proposes to make KI sections mandatory for all FDA-regulated research [11].

Neither the Common Rule nor FDA’s proposed rule enumerates the specific content or format of KI sections [7,10]. The preamble to the final version of the revised Common Rule, however, does suggest several possible topics for inclusion: “(1) the fact that consent is being sought for research and that participation is voluntary; (2) the purposes of the research, the expected duration of the prospective subject’s participation, and the procedures to be followed in the research; (3) the reasonably foreseeable risks or discomforts to the prospective subject; (4) the benefits to the prospective subject or to others that may reasonably be expected from the research; and (5) appropriate alternative procedures or courses of treatment, if any, that might be advantageous to the prospective subject” [12]. These topics represent a subset of the nine basic elements of informed consent required by the Common Rule [10,12]. The preamble also makes clear that information considered to be “key” for any given research study will be study-specific and subject to requirements set by the sponsoring institution or institutional review board (IRB) [12]. Further, in 2018, the US Department of Health and Human Services (HHS) Secretary’s Advisory Committee on Human Research Protections (SACHRP) offered a list of questions to help determine the content of KI sections [13], although these questions explicitly were not designed to be either comprehensive or to “be used as a checklist” [13]. Others have also considered what topics should be included in KI sections [14–17].

In addition to informational content, the revised Common Rule states that the KI section “must be organized and presented in a way that facilitates comprehension” [10]. It does not, however, provide detailed instruction on how to accomplish this [12]. Prior work has documented variability in the content and format of KI sections. Mozersky and colleagues [18] have reviewed consent templates and guidance documents from the IRB websites of 46 US medical schools. While the authors identified 36 unique KI topics, they found that the majority of institutions recommended including topics identified in the preamble to the revised Common Rule [18]. The authors also found that only 41% of the institutional guidance documents recommended using plain language [18]. Solomon and colleagues [19] surveyed 232 principal investigators and 1052 clinical research coordinators on their experience with ICFs containing KI sections over a 12-month period. Their respondents reported that 63% of ICFs used plain language and 42% used at least 12-point font and were formatted with increased white space [19]. Further, plain language and formatting were identified as the two main practices for communicating health information in ways that facilitate comprehension.

While previous work has surveyed guidance, recommendations, and practices, actual KI sections from IRB-approved ICFs used in clinical trials have not been systematically characterized. Since January 21, 2019, completed trials subject to the revised Common Rule must have an ICF posted on ClinicalTrials.gov or in a docket folder on Regulations.gov [10,20]. Access to a broad set of these documents provides opportunities for research on current practices across multiple trials, sponsors, and organizations, as well as the development of best practices [21]. Here, we characterized a sample of ICFs posted on ClinicalTrials.gov. We first characterized key trial attributes and then analyzed page length, word count, readability, topics, and formatting of KI sections and full ICFs.

The motivation for this project stems from the role that analyses of existing KI sections can play in empowering the research community to fulfill the regulatory requirement for KI sections in ways that enhance the comprehensibility of informed consent documents and improve the informed consent process generally. While KI sections present an opportunity to facilitate effective informed consent for research participation, the final version of the revised Common Rule did not provide actionable guidance concerning the content or format of KI sections. Further, researchers seeking to create well-designed KI sections will find a paucity of evidence-based practice guidelines. The research needed to build an adequate evidence base for how to structure and use KI sections to improve communication and comprehension, and to support decision-making, will require time. The requirement for KI sections is, however, already in place, creating a gap in which research stakeholders are likely to struggle with the KI section requirement. Our analysis, which evaluates existing KI sections for trends and gaps in current practice, helps to inform the development of KI sections (e.g., providing support for PIs in drafting and IRBs in reviewing KI sections) before more formal guidance is available, as well as help to identify priorities for future research and guideline development.

## Materials and methods

### Data collection

We searched ClinicalTrials.gov for registered clinical trials funded by at least one US Federal agency listing “treatment” as the primary purpose of the intervention(s) being evaluated, a study initiation date of January 21, 2019, or later, and first posted on ClinicalTrials.gov on or before October 1, 2021 (see S1 Appendix for search terms). These Reference Sample records were retrieved on April 21, 2022.

We then searched ClinicalTrials.gov using the same search terms with the additional criterion for records with posted ICF documents (see S2 Appendix for search terms). These Study Sample records, which were retrieved on October 1, 2021, consisted of a subset of Reference Sample records with posted ICFs. The posted ICF for each study in the Study Sample was downloaded. For the nine studies that had more than one ICF posted on ClinicalTrials.gov, one coder (WM) chose one ICF for further analysis based on a predetermined selection method (S1 Table).

### Data analysis

Study records from the Study and Reference Samples were characterized by the following attributes: intervention type, study phase, recruitment status, and enrollment size. Studies labeled with more than one intervention type were assigned to multiple intervention type categories.

We developed a codebook in Microsoft Excel to analyze the ICFs and KI sections in the Study Sample. A list of KI topics was adapted from Mozersky *et al*. [18], the preamble to the revised Common Rule [12], 45 C.F.R. § 46.116 [10], and the 2018 SACHRP Commentary [13]. In addition, a list of formatting elements was taken from Mozersky *et al*. [18], to which we added additional elements identified from a review of the ICFs in the Reference Sample prior to coding (see S2 Table). Two coders (AG and WM) independently piloted the codebook on five ICFs from the Study Sample and revised the codebook to improve the clarity of the instructions. Based on subsequent independent coding of another five ICFs from the Study Sample, the coders achieved an interrater reliability of 98%. The remaining ICFs in the Study Sample were analyzed using the final codebook in batches of approximately 25 ICFs. For each batch, AG analyzed all ICFs, and WM used a random number generator to select five of the ICFs to code and compare for consistency. Intercoder reliability was 94% (582/616). Discrepancies in coding were discussed and resolved by a study team member (BEB).

Coders recorded whether a KI section was present in each ICF in the Study Sample, and, if so, which KI topics and formatting elements were used. KI sections were analyzed by length and readability and summarized with descriptive statistics (mean with range and median with interquartile range or IQR).

The length of KIs and full ICF documents (i.e., including the KI and all other ICF sections) was characterized both by the number of pages (rounded to the nearest quarter page) and word count (without headers and footers). We also calculated the ratio of KI section length to the length of the full ICF in which they appeared in terms of both measures. KI sections and ICFs were characterized by readability in terms of grade level, calculated using (up to) the first 3000 words of each ICF with the SMOG Index [22], which has been shown to be the most consistent readability standard for evaluating health materials [23]. KI sections and ICFs were considered to be written at the same grade level if the leftmost digit(s) was the same and a different grade level if the leftmost digit(s) was different; for example, SMOG scores of 8.1 and 8.7 would both indicate an 8th-grade reading level (and so on for other grade levels), despite the difference after the decimal. This is based on the assumption, reflected in the SMOG Index, that there is a range of reading ability within each grade level. Differences in SMOG grade levels between KI sections and corresponding ICFs were assessed and further characterized by intervention type.

## Results

### Sample trial characteristics

Of 102 trial records with posted ICF documents in the Study Sample (Table 1), 76 (75%) of the ICFs included a readily identifiable KI section. Based on a comparison of attributes between these 76 trials in the Study Sample and the 2,535 trials in the Reference Sample, we identified several notable differences, including differences in the proportion of Phase 2/3 or 3 studies, enrollment status, and the proportion of studies with an actual or estimated sample size greater than 500. Additional sample characteristics are found in Table 1.

**Table 1.** Characteristics of trials in the study and reference samples

Trial attribute	Study sample (*n* = 102 trials)		Reference sample (*n* = 2,535 trials)
	Studies with ICFs and KI section (*n* = 76 trials)	Studies with ICFs and No KI section (*n* = 26 trials)	
	No. (%)	No. (%)	No. (%)
**Intervention type –** At least one of the following			
Drug, biologic, or genetic	40 (53)	9 (35)	1391 (55)
Early Phase 1 or Phase 1	8 (20)	0 (0)	341 (25)
Phase 1/2 or Phase 2	18 (45)	7 (78)	776 (56)
Phase 2/3 or Phase 3	10 (25)	1 (11)	172 (12)
Phase 4	4 (10)	1 (11)	80 (5.8)
Phase listed as “Not Applicable”	0 (0)	0 (0)	22 (1.6)
Behavioral	23 (30)	13 (50)	697 (27)
Device, diagnostic, medical procedure, or radiation	15 (20)	2 (7.7)	634 (25)
Combination product, dietary supplement, or other	21 (27)	6 (23)	628 (25)
**Overall recruitment status**			
Not yet recruiting; enrolling by invitation; recruiting; or active, not recruiting	43 (57)	11 (42)	2108 (83)
Completed; suspended; terminated; or withdrawn	33 (43)	15 (57)	398 (16)
Unknown	0 (0)	0 (0)	29 (1.1)
**Enrollment (anticipated or actual)**			
0–50 participants	26 (34)	13 (50)	1136 (45)
51–100 participants	16 (21)	5 (19)	514 (20)
101–500 participants	22 (29)	6 (23)	722 (28)
>500 participants	12 (16)	2 (7.7)	163 (6.4)

### Page length

**Figure 1.**
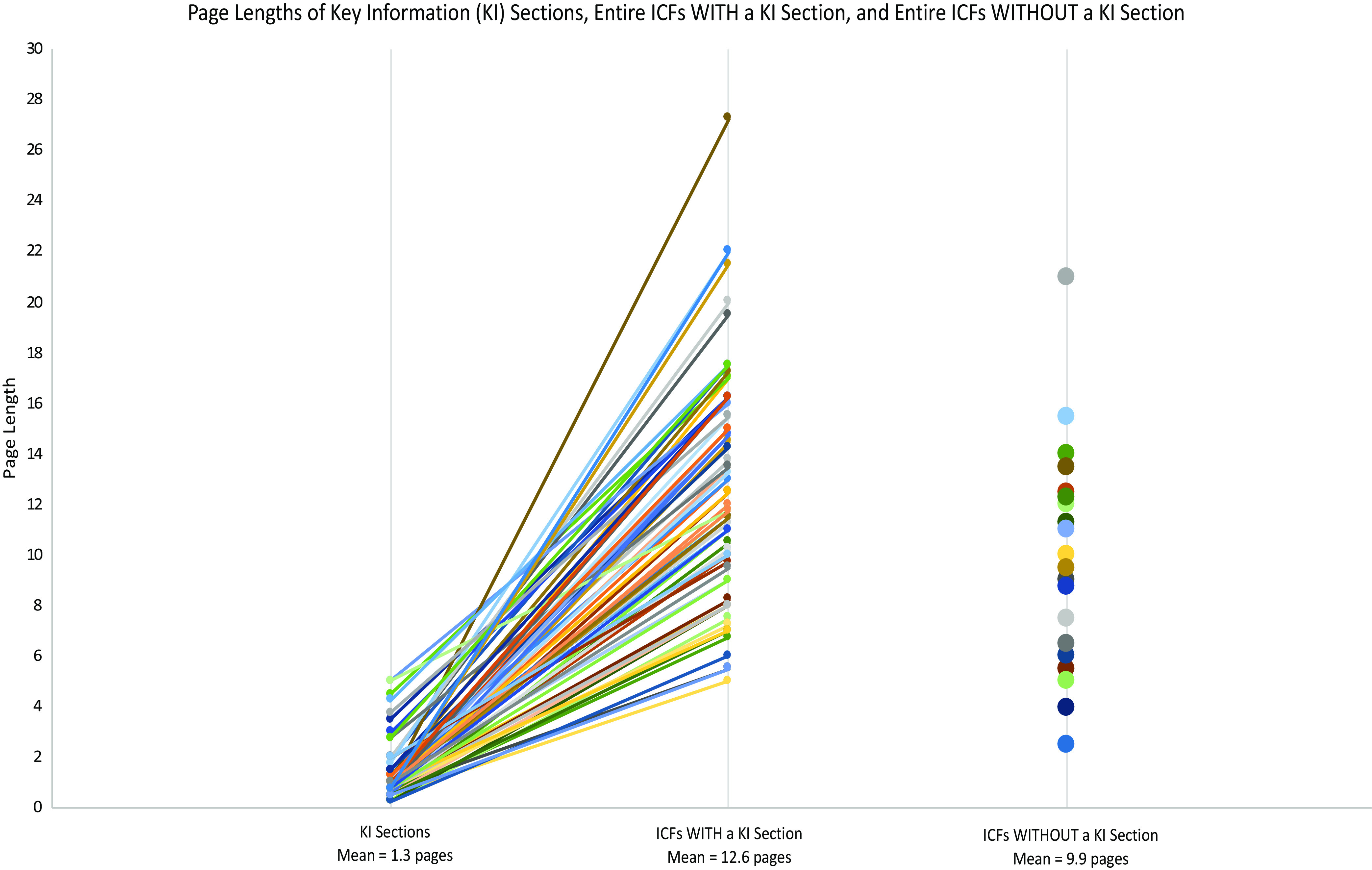
Page lengths of key information (KI) sections and informed consent forms (ICFs). For each ICF with a KI section, a line is drawn between the KI section data point and the corresponding ICF data point.

Overall, the page length of ICFs with KI sections (mean 12.6, range 5–27.5 pages) was longer than ICFs without them (mean 9.9, range 2.5–21 pages), with a mean difference of 2.7 pages. The mean page length of KI sections was 1.3 pages (range 0.25–5 pages) which, on average, constituted 10% of the total page length of ICF documents (median 8.4%, IQR, 6.2–11%). Page lengths of KI sections and their corresponding ICFs were not correlated (Fig. 1 and S1 Figure). That is, longer KI sections did not generally predict longer overall ICFs, and shorter KI sections did not generally predict shorter ICFs. When length was measured by word count, the findings were similar (S3 Appendix), which is unsurprising given the general absence from KIs of non-narrative graphic representations (see below).

### Readability

**Figure 2.**
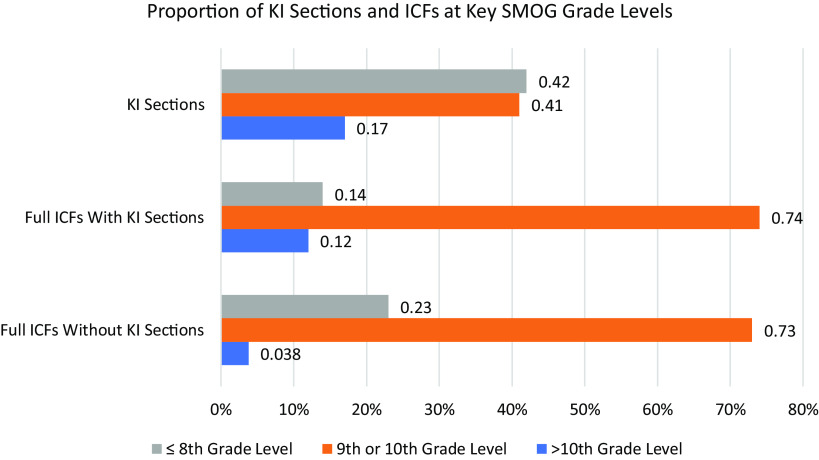
Proportions of key information (KI) sections and informed consent forms (ICFs) at key SMOG grade levels. (A) Gray bars represent the proportion of documents written at or below an 8th-grade SMOG reading level. (B) Orange bars represent the proportion of documents written at a 9th- or 10th-grade SMOG reading level. (C) Blue bars represent the proportion of documents written above a 10th-grade SMOG reading level.

**Figure 3.**
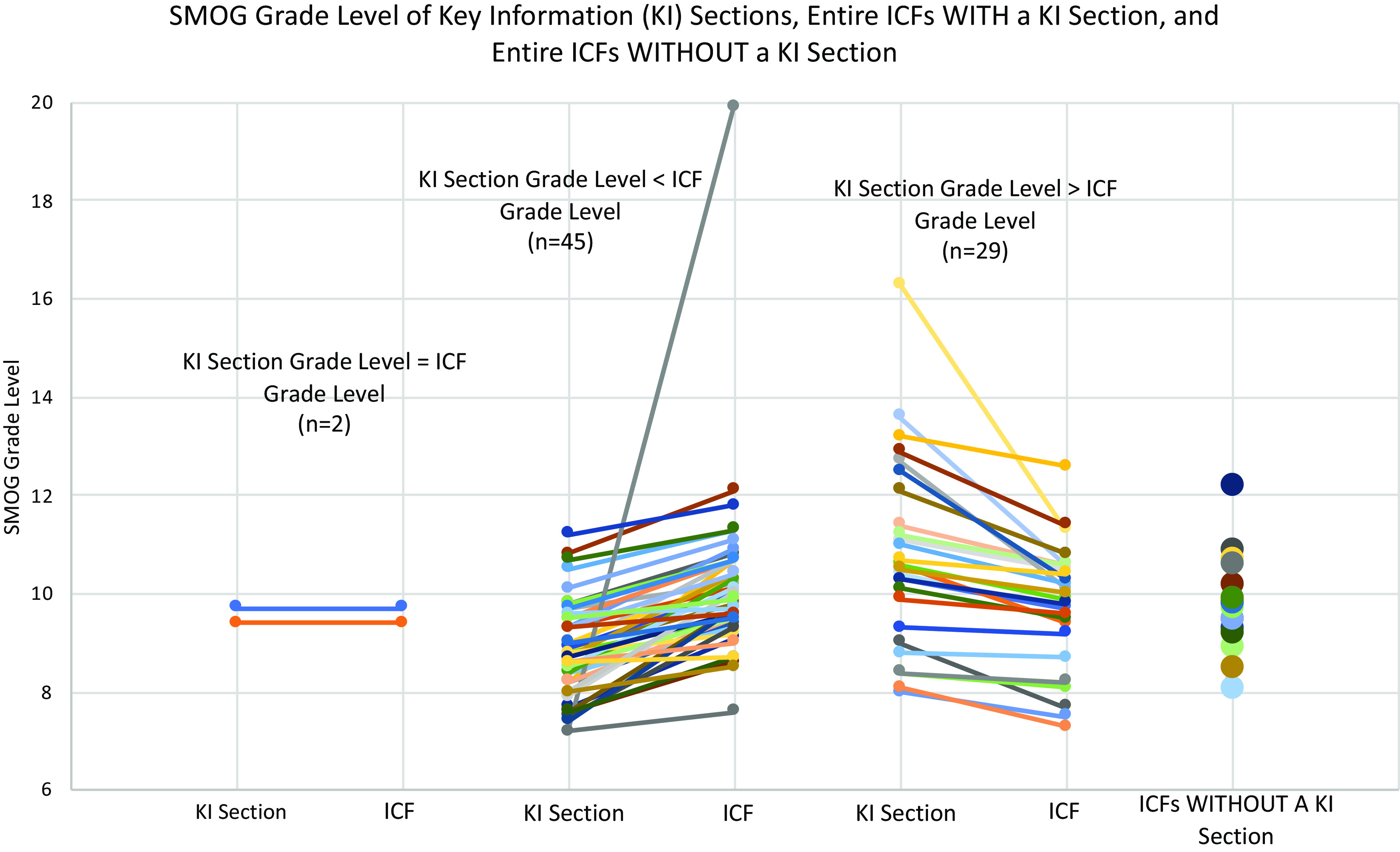
SMOG readability grade levels of key information (KI) sections and informed consent form (ICFs). For each ICF with a KI section, a line is drawn between the KI section data point and its corresponding ICF data point.

The mean grade level by SMOG Index for KI sections was 9.6, with a range of 7.2–16.3 grade levels. There was very little difference in the SMOG Index between full ICFs that included a KI section (mean 10, range 7.3–19.9 grade level) and those that did not (mean 9.7, range 8.1–12.2) (S3 Table). Similarly, there was little difference between the mean readability of KI sections themselves (9.6 grade level) and full ICFs, either with (10.0 grade level) or without (9.7 grade level) a KI section (S3 Table). At the same time, the proportion of KI sections readable at or below the 8th grade level (32/76, 42%), which is often taken as a goal for ICF readability [24–27], was notably higher than the proportion of full ICFs readable at the same level (11/76, 14%) (Fig. 2). In other words, while KI sections on average were not much more readable than full ICFs, many more of them met a common standard for readability. When we compared KI sections to the ICFs in which they appeared, approximately half of the KI sections (37/76, 49%) were more readable than the full ICF, while 28% (21/76) were less readable (with the remainder being assessed at the same grade level) (Fig. 3).

### KI topics

A majority of KI sections in the Study Sample included topics suggested in the preamble to the revised Common Rule (Table 2). We identified several notable differences among the topics included in KI sections by trial intervention type (Table 2). For example, the presence in KI sections of an explicit statement that research participation is voluntary varied between ICFs for trials involving behavioral interventions (100%) and trials involving drugs, biologics, or genetics (83%), combination products (81%), and devices (80%). The presence in KI sections of a statement about how participation may differ from treatment outside the study differed between trials involving drugs, biologics, or genetics (88%) and trials involving combination products (71%), devices (60%), and behavioral interventions (39%). And the presence in KI sections of a description of the procedures to be followed varied between ICFs for trials involving drugs, biologics, and genetics (90%) and trials involving behavioral interventions (65%), among others. Strikingly, a substantial minority of KI sections did not state the main reasons to join a study (20%) or the main reasons *not* to join a study (14%), with this varying across study types (Table 2).

**Table 2.** Key information (KI) topics included in informed consent forms (ICFs) by intervention type^a,b^

KI topic	Intervention type				
	Total ICFs (*n* = 76)	Drug, biologic, or genetic (*n* = 40)	Behavioral (*n* = 23)	Device, diagnostic, medical procedure, or radiation (*n* = 15)	Combination product, dietary supplement, or other (*n* = 21)
The purpose of the research, the study’s research questions, or why the study is relevant for the participant^C,P,S^	76 (100)	40 (100)	23 (100)	15 (100)	21 (100)
The expected duration of research participation^C,P^	68 (89)	37 (93)	19 (83)	15 (100)	18 (86)
The procedures (or types of activities) to be followed in the research^C,P,S^	61 (80)	36 (90)	15 (65)	12 (80)	17 (81)
The main reasons not to join the study, including reasonably foreseeable risks or discomforts to the prospective participant^C,P,S^	65 (86)	35 (88)	20 (87)	13 (87)	17 (81)
The main reasons to join the study, including benefits to the prospective participant or others^C,P,S^	61 (80)	34 (85)	20 (87)	12 (80)	16 (76)
Alternative procedures or courses of treatment^C,P^	51 (67)	27 (68)	16 (70)	11 (73)	15 (71)
Confidentiality^C^	19 (25)	7 (18)	9 (39)	4 (27)	4 (19)
Whether compensation and/or medical treatments are available if injury occurs^C^	0 (0)	0 (0)	0 (0)	0 (0)	0 (0)
Whom to contact for questions or in case of injury^C^	37 (49)	23 (58)	11 (48)	7 (47)	10 (48)
A statement that research participation is voluntary^C,P^	65 (86)	33 (83)	23 (100)	12 (80)	17 (81)
Information on data or biospecimen use for future research^C^	5 (6.6)	4 (10)	0 (0)	1 (6.7)	2 (9.5)
What will differ from treatment outside the study; what will be unfamiliar to the participant, unexpected, or require special attention^S^	50 (66)	35 (88)	9 (39)	9 (60)	15 (71)
What information is being collected from the participant^S^	23 (30)	13 (33)	6 (26)	2 (13)	10 (48)
Payment for participation	10 (13)	8 (20)	3 (13)	0 (0)	4 (19)

^a^Topics are listed by the order in which they appear in the Common Rule Basic Elements of Informed Consent, annotated as “C.” Topics not in the Common Rule Basic Elements of Informed Consent are ordered by the frequency with which they were observed in KI sections in the total sample. “P” indicates the topic is mentioned in the Potential Elements of KI from the preamble to the Revised Common Rule. “S” indicates the topic is mentioned in SACHRP’s Commentary on the New KI Informed Consent Requirements.

^b^If a study was labeled with more than one intervention type, it was counted in each of its intervention type categories.

### Formatting

All (76/76, 100%) KI sections in the Study Sample used narrative summary (Table 3). Of the other coded formatting elements, most KI sections were distinguished from the rest of the ICF by a section header (70/76, 92%). Of the other coded formatting elements, only bulleted, numbered, or lettered lists (40/76, 53%) were used in a majority of KI sections. Few KI sections used text boxes (23/76, 30%) or tables (5/76, 6.6%), and no KI section used images or graphics. On average, KI sections with sub-headers or lists were approximately double the page length and word count of those without (Table 4). See S2 Table for a fuller description of the different formatting elements.

**Table 3.** Formatting elements in informed consent forms (ICFs) with a key information (KI) section (*n* = 76)^a,b^

Formatting element	Intervention type no. of records (% total)				
	Total ICFs (*n* = 76)	Drug, biologic, or genetic (*n* = 40)	Behavioral (*n* = 23)	Device, diagnostic, medical procedure, or radiation (*n* = 15)	Combination product, dietary supplement, or other (*n* = 21)
Narrative summary	76 (100)	40 (100)	23 (100)	15 (100)	21 (100)
Section header	70 (92)	37 (92.5)	22 (96)	14 (93)	19 (90)
Bulleted, numbered, or lettered list^M^	40 (53)	24 (60)	8 (35)	9 (60)	14 (67)
Subheaders	30 (39)	20 (50)	6 (26)	3 (20)	14 (67)
Q&A format	16/30 (53)	11/20 (55)	2/6 (33)	2/3 (67)	9/14 (64)
Other format	14/30 (47)	9/20 (45)	4/6 (67)	1/3 (33)	5/14 (36)
Text box and/or shaded background	23 (30)	13 (33)	7 (30)	5 (33)	4 (19)
Text font(s)^M^	6 (7.9)	1 (2.5)	1 (4.3)	3 (20)	1 (4.8)
Tables^M^	5 (6.6)	5 (13)	0 (0)	0 (0)	2 (9.5)
Color	1 (1.3)	1 (2.5)	0 (0)	0 (0)	0 (0)
Images or graphics^M^	0 (0)	0 (0)	0 (0)	0 (0)	0 (0)

^a^Elements are ordered by the frequency with which they were observed in key information (KI) sections in informed consent forms (ICFs) in the Study Sample. Elements annotated with “M” were adapted from Mozersky and colleagues.

^b^If a study was labeled with more than one intervention type, it was counted in each of its intervention type categories.

**Table 4.** Page lengths and word counts of key information (KI) sections by use of sub-headers and lists

	Use of sub-headers		Use of bulleted, numbered, or lettered list	
	Used (*n* = 30)	Not used (*n* = 46)	Used (*n* = 40)	Not used (*n* = 36)
Page length				
Mean (range)	1.9 (0.5–5)	0.91 (0.25–5)	1.7 (0.5–5)	0.84 (0.25–5)
Median (IQR)	1.4 (1–2.6)	0.75 (0.5–1)	1.3 (1–2)	0.75 (0.5–1)
Word count				
Mean (range)	804 (176–2170)	422 (131–985)	746 (262–2170)	381 (131–863)
Median (IQR)	577 (445–873)	365 (285–520)	530 (417–890)	343 (269–471)

## Discussion

The revised Common Rule permits broad flexibility in the presentation and content of KI sections, requiring only that KI sections be concise and focused, facilitate comprehension, and contain information material to decision-making [10]. We identified 102 trials with posted ICFs from a total of 2,535 registered trials that appear to fall under the revised Common Rule (Table 1). Of those, 75% (76) included a readily identifiable KI section. There were several notable differences among attributes of trials in the Study and Reference Samples (Table 1). While over half of Sampled trials that had ICFs with KI sections (53%), and over half the trials in the Reference Sample (55%), involved drug, biologic, or genetic interventions, only approximately a third (35%) of Sampled ICFs without KI sections included any of these intervention types. By contrast, the proportions were reversed with respect to use of behavioral interventions: half (50%) of Sampled trials that had ICFs without a KI section listed them, but only 30 and 27% of Sampled trials with a KI section and Reference sample trials, respectively. And among large trials enrolling 500 or more participants, the proportion of Sampled trials that had ICFs with KI sections (16%) was more than twice those of Sampled trials without KI sections (7.7%) and Reference trials (6.4%).

In terms of the text length, KI sections in the Study Sample were generally “concise and focused” [10] and relatively short [8]: a mean length of 1.3 pages and comprising, on average, 10% of the total page length of the ICFs in which they appear (Fig. 1). Further, none of the sampled KI sections exceeded five pages, which is significantly shorter than the “10-page description” given as an example of an unacceptably long KI section in the preamble to the revised Common Rule [8].

Although the revised Common Rule does not specify what it means for KI sections to be “organized and presented in a way that facilitates comprehension” [10], Solomon and colleagues [18] identified the use of plain language and optimal formatting as the main ways to fulfill this requirement. On average, we detected no notable difference in average readability between KI sections and ICFs overall (S3 Table), which is consistent with previous work [28] and suggests that KI sections are not generally receiving more attention to improved readability than other parts of the ICF. Indeed, we found that most KI sections in the Study Sample failed to meet an 8th grade or lower readability standard (Fig. 2), a common expectation for ICFs [29–32]. This strongly suggests that the readability of many KI sections could be improved.

The single substantive criterion for KI sections in the revised Common Rule states that KI sections must present the “information that is most likely to assist a prospective subject […] in understanding […] why one might or might not want to participate in the research” [10]. Our analysis of KI sections by topic showed that 20% of sampled KI sections failed to include a discussion of the main reasons in favor of participating and 14% failed to include a discussion of the main reasons *not* to participate (Table 2). These reasons include the risks and benefits of study participation. From a normative perspective, it seems plausible that *all* KI sections should at least briefly cover the risks and benefits of participation, regardless of study design or population.

Best practices for formatting health materials, which can assist in comprehension by rendering complex information easier to understand, include separating sections using headers, highlighting important text with bullet points or bolding [18,33–37] and displaying information in tables, charts, and images [33]. Over 90% of ICFs with KI sections in the Study Sample distinguished the KI section with a section header and over half used lists, but only a minority used sub-headers and few used images, tables, or text boxes (Table 3). While we believe that the use of more formatting elements in KI sections would improve understanding, we acknowledge that organizing information using formatting elements often takes up more lines on a page than the equivalent narrative format [19]. This may explain, at least in part, why KI sections in the Study Sample with lists or sub-headers were approximately twice the page length and word count of those without such elements (Table 4). However, in our view, the potential for formatting elements to add length is a reasonable trade-off in the service of understandability.

That said, more research is needed to understand how the content and formatting for KI sections can optimally address the needs of potential participants as envisioned by the Common Rule. Ultimately, synthesizing such evidence in regulatory guidance would help the research community adopt best practices to ensure that the information presented is clear and concise across KI sections, but with sufficient flexibility to adjust for specific research situations. An example can be found in FDA guidance on patient labeling for human prescription drug and biological products, which provides recommendations on document readability as well as style and layout with references to the literature [38,39].

In its commentary on the KI requirement, SACHRP raised the concern that the potential KI topics suggested in the preamble to the revised Common Rule could become “a safe harbor of sorts” and ultimately undermine the intent of the KI requirement “to enable institutions and individuals to tailor informed consents to the circumstances of particular studies” [13]. While we were able to conclude that KI sections are primarily drawing from the topics suggested in the preamble (Table 2) [12], which is concordant with other research [18], we did not find strong evidence that SACHRP’s worry has eventuated. Indeed, our results support the idea that KI sections are being tailored in study-specific ways, as we found numerous differences between the topics included in KI sections, depending on the intervention type under evaluation in the study (Table 2). It is helpful here to keep in mind that “intervention types” correspond to different communities and sub-communities of researchers and research, each of which have distinct requirements, norms, and practices, which may, in turn, be reflected in the content of KI sections. For instance, trials of drugs, biologics, or generics in the USA are subject to FDA regulatory requirements. Further, many KI sections included topics that are not suggested in the preamble to the revised Common Rule at all. For example, two-thirds of studies with KI sections included a discussion of how the treatment offered inside the study differs from treatment outside it, as suggested by SACHRP guidance but not by the preamble to the Common Rule (Table 2) [12,13].

While many of the sampled KI sections in our analysis aligned with the few expectations specified in the preamble to the revised Common Rule, we also identified areas in which KIs could be significantly improved. Although many have called for the issuance of formal regulatory guidance to clarify the regulatory requirements for KI sections [7,16,40], more systematic research is needed to inform development of such guidance. In the short term, the research community should strive toward consensus-based guidelines to support the development of effective and understandable KI sections. For instance, the several axes analyzed in this study are linked to established guidelines for writing more understandable KI sections, such as using plain language [41] to improve comprehension for readers at all levels of health literacy [42]. Indeed one general focus of guidelines could be to improve the comprehensibility of KI sections by recommending maximum page lengths, expectations for maximum grade-level readability, plain language, and formatting elements [10], such as sub-headers and lists. In addition, with respect to the content of KI sections, regulatory text already mentions the reasons for and against participation as topics for inclusion in KI sections; guidelines could further stress the importance of including key risks and benefits of participation, given the rate at which this information failed to be included in the KI sections we sampled. For therapeutic trials, guidelines could also stress the importance of including alternative therapies or courses of treatment available outside the trial. Such guidelines generally could be presented as recommended defaults, subject to a rebuttable presumption for strong countervailing reasons. Further, checklists could be produced to help researchers determine when it is appropriate to depart from the default (e.g., omitting “alternative procedures or courses of treatment” for healthy volunteer studies).

In the absence of specific requirements in the revised Common Rule, research institutions currently produce their own norms and guidance, including recommending various page lengths for KI sections: “a single short paragraph, 2–3 paragraphs, no more than half or one page, 2–3 pages, a few pages, and in one case […] no more than 1/3 the length of the entire consent form” [18]. Such institutional policies may be more determinate and less flexible than is optimal, limiting the promise and value of KI sections. In addition to promoting consistency and shared standards between institutions, guidelines and clarification now could also help optimize harmonization efforts between FDA regulations and the Common Rule, should FDA’s proposal for a similar requirement for KI sections be adopted.

Widely adopted guidelines could also facilitate compliance analyses that are currently challenged by the broad subjectivity in interpreting the KI requirement. Even determining whether an ICF contains a KI section in compliance with the revised Common Rule is complicated by the provision in the preamble that when an ICF is “relatively brief […] an institution may determine that” the ICF itself satisfies the KI requirement [12]. Among the quarter (26/102) of ICFs without a readily identifiable KI section in our sample, 24 of the associated trial records listed a Common Rule signatory as a funder. Of those 24 studies, 18 ICFs were greater than 6 pages in length, which may suggest that some institutions take a permissive approach to what counts as brief enough to waive the KI requirement [18].

### Limitations

Our sample is not representative of the full universe of ICFs and potential KIs, since it was restricted to federally funded studies, and industry-funded and other studies may include KI sections even when they are not required by regulations. We intend this as an exploratory study, given the small sample sizes based on the limited availability of ICF documents with a clearly marked KI section for trials initiated after the revised Common Rule effective date (January 2019 onward), yielding less than 3 years of data. Given the relatively small number of ICFs with KI sections available on ClinicalTrials.gov, we were not confident that our sample would be representative; we did not, therefore, prespecify null hypotheses for testing nor employ inferential statistics. Larger sample sizes in the future would be needed to confirm the findings reported here. We also note that the promulgation of FDA’s proposal to require KI sections for FDA-regulated research will permit the study of these matters more broadly.

Finally, assessing whether KI sections accomplish certain aims—for example, disclosing information that *accurately* summarizes possible reasons in favor of or against participating—would require assessments of further details about the study, and/or features of the ICF, that we did not perform. More basically, there is a lack of robust empirical data on what does or does not assist participant understanding during the research informed consent process, which makes it difficult to be confident about the extent to which KI sections are successful in their chief purpose, namely, facilitating better participant understanding and improved decision-making. More empirical work is needed on the factors conducive to understanding during informed consent for research and the drivers of decision-making about research participation.

### Looking ahead

While calls for formal regulatory guidance are understandable given the ongoing uncertainty faced by the regulated community in trying to comply with the KI provision, deeper examination of underlying challenges for reaching the goal of “concise and focused” KI sections is needed. In reviewing actual KI sections from ICFs for federally funded trials initiated after the effective date of the revised Common Rule, our findings not only confirm prior work on KI sections [43] but go well beyond those analyses to characterize the conciseness and content of KI sections using several additional measures that are relevant to adjudicating their overall quality. Still, nearly 5 years after the KI requirements became effective, there is a need for more research, which will be facilitated by the ever-increasing number of publicly available consent forms and the more sophisticated analyses larger sample sizes make possible.

Importantly, however, restricting analyses to the properties of consent forms is insufficient. Consideration must also be given to the preferences and behavior of potential participants and the environment in which this information is presented, given the maxim that informed consent is not a form but a *process*. Example of such broader research questions might include:

1. How do we ensure that the information presented in the KI section is consistent with the full ICF, but considerably shorter and easier to understand?
2. Under what conditions, or to what extent, do and should items that appear in the KI section also appear in other sections of the ICF?
3. To what extent do participants in different contexts depend on the KI section to the exclusion of other sections?
4. To what extent does the information provided in the KI section represent accurately (and completely) what potential participants in different contexts would need to consider before joining a study?

Rigorous research into such questions can help focus future regulatory guidance and ultimately assist the research community in ensuring that KI sections fulfill their goal and, ultimately, the high calling of assisting individuals in making good and well-informed decisions about whether to participate in research.

## Supporting information

Gelinas et al. supplementary material Gelinas et al. supplementary material

## Supplementary material

The supplementary material for this article can be found at https://doi.org/10.1017/cts.2023.605.
