# Supplementary material for: Characterization of key information sections in informed consent forms posted on ClinicalTrials.gov
Source: J Clin Transl Sci. 2023 Aug 14;7(1):e185. doi: 10.1017/cts.2023.605 (PMC10514692; doi:10.1017/cts.2023.605)
Supplement: Gelinas et al. supplementary material [file S2059866123006052sup001.docx]

**Supplementary Information**

**S1 Appendix. ClinicalTrials.gov Search Terms for the Reference Sample.**

AREA[DesignPrimaryPurpose] "Treatment" AND AREA[StudyType] "Interventional" AND AREA[FunderTypeSearch] ( "NIH" OR "FED" ) AND AREA[StartDate] RANGE[01/21/2019, MAX] AND AREA[StudyFirstPostDate] RANGE[MIN, 10/01/2021]

**S2 Appendix. ClinicalTrials.gov Search Terms for the Study Sample.**

AREA[LargeDocHasICF] "Yes" AND AREA[DesignPrimaryPurpose] "Treatment" AND AREA[StudyType] "Interventional" AND AREA[FunderTypeSearch] ( "NIH" OR "FED" ) AND AREA[StartDate] RANGE[01/21/2019, MAX]

**S1 Table. Eligibility Criteria for Study Records with Multiple Posted ICFs.**

If multiple versions of an ICF tailored for different locations were posted for a single trial, the ICF for the study location most closely associated with trial sponsor listed on ClinicalTrials.gov was selected (e.g., “Bethesda, Maryland, US” for NIH).

| **Type of ICF Pairs** | **ICF Included for Study** | **ICF Excluded from Study** |
| --- | --- | --- |
| Donor and. Recipient | For individuals receiving the biological material | For individuals donating biological material for use in the study |
| Screening and Research | For the research part of the study | For the screening part of a study |
| Main Study and Sub-Study | For the main study | For a sub-study |
| Main Study and Future Use | For the main study | For the future use of data and biospecimens |
| Main Study and Specific Intervention | For the main study | For the specific intervention |
| Tailored for Study Locations | For the study location most closely associated with trial sponsor listed on ClinicalTrials.gov (e.g., “Bethesda, Maryland, US” for NIH) | For all other study locations |

**S2 Table. KI Formatting Elements.**

Elements annotated with “M” were adapted from Mozersky and colleagues [18]. Other elements were identified by the research team review of ICFs in the Reference Sample but not included in the Study Sample prior to coding.

| **Formatting Element** | **Description** |
| --- | --- |
| Section header | Label to identify the KI section (e.g., “Key Information”) |
| Text box and/or shaded background | Use of a callout box around text and/or highlighted background to highlight the KI section |
| Narrative summary | Paragraph(s) or set of sentences |
| Subheaders | Use of word or phrases to separate sentences/narrative text (e.g., “Purpose”) |
| *Variation 1*: Q&A format | Use of questions as subheaders (e.g., “What is this research about?”) |
| *Variation 2*: Structured data elements | A subheader followed by data or brief phrases rather than sentences/narrative text (e.g., “**Participation Duration**: 6 months”) |
| Bulleted, numbered, or lettered list^M^ | Use of bulleted, numbered, or lettered text |
| Color | Use of colored font(s) |
| Text font(s)^M^ | Use of different font designs including **bold**, *italics*, ALL CAPS, underline, and/or larger font size than other text |
| Images or graphics^M^ | Use of images to illustrate key concepts (e.g., infographics) |
| Tables^M^ | Use of tables to illustrate key concepts |

**S1 Figure. Page Lengths of KI Sections vs. ICFs (n=76).**

**S3 Appendix. Word Count Results.**

Mean word counts of ICFs with KI sections (n=76; mean 5126 words, range 2189-10444 words) were longer than ICFs without KI sections (n=26; mean 4113 words, range 938-8563 words), with a mean difference of 841 words. The mean word count of KI sections (n=76) was 573 words (range 131-2170 words). On average, the KI sections constituted 12% of the total word count of those ICF documents that contained KIs, with a median of 10% (IQR 6.5%-14%).

**S3 Table. SMOG Index Grade Level of KI Sections and ICFs.**

|  | **KI Sections  (n=76)** | **Full ICFs With KI Sections (n=76)** | **Full ICFs Without KI Sections (n=26)** |
| --- | --- | --- | --- |
| Mean (Range) Grade Level | 9.6 (7.2-16.3) | 10 (7.3-19.9) | 9.7 (8.1-12.2) |
| Median (IQR) Grade Level | 9.3 (8.4-10.5) | 9.9 (9.4-10.6) | 9.8 (9.2-10) |
